# Short-Lived Exercise-Induced Exerkines Modulate Inflammation for Chronic Disease Prevention: A Systematic Review and Meta-Analysis

**DOI:** 10.3390/biom15111590

**Published:** 2025-11-13

**Authors:** Hossein Poorhabibi, Katja Weiss, Thomas Rosemann, Beat Knechtle, Rasoul Eslami, Bakhtyar Tartibian, Seyed Morteza Tayebi, Rahman Sheikhhoseini

**Affiliations:** 1 Department of Exercise Physiology, Faculty of Physical Education and Sports Sciences, Allameh Tabataba’i University, Tehran 1485643449, Iran; poorhabibih@gmail.com (H.P.); ba.tartibian@gmail.com (B.T.); tayebism@atu.ac.ir (S.M.T.); 2 Institute of Primary Care, University Hospital of Zurich, 8091 Zurich, Switzerland; katja@weiss.co.com (K.W.); thomas.rosemann@usz.ch (T.R.); 3 Department of Sport Injuries and Corrective Exercises, Faculty of Physical Education and Sport Science, Allameh Tabataba’i University, Tehran 1485643449, Iran; rahman.pt82@gmail.com

**Keywords:** exerkines, exercise, inflammation, chronic disease, interleukin-6, tumor necrosis factor-alpha, interleukin-10, C-reactive protein

## Abstract

Physical exercise triggers the release of short-lived exerkines, such as interleukin-6 (IL-6), tumor necrosis factor-alpha (TNF-α), and interleukin-10 (IL-10), which may help reduce systemic inflammation and mitigate the risk of chronic disease. Despite their potential, the effects of these exercise-induced cytokines (termed exerkines) across diverse populations remain underexplored. This study evaluated how exercise-induced exerkines modulate inflammatory markers, based on changes observed before and after intervention. We systematically searched PubMed, Scopus, and Web of Science from January 2015 up to 7 February 2025, identifying 11 randomized controlled trials (RCTs) involving 1135 participants. Standardized mean differences (SMDs) with 95% confidence intervals (CIs) were calculated using a random-effects model to assess changes in IL-6, TNF-α, IL-10, C-reactive protein (CRP), and interferon-gamma (IFN-γ). Study quality was evaluated using the Cochrane Risk of Bias 2 tool. Exercise significantly reduced CRP (SMD = −0.77, 95% CI: −1.20 to −0.33, *p* = 0.001) and TNF-α (SMD = −1.09, 95% CI: −2.14 to −0.03, *p* = 0.043) while increasing IL-6 (SMD = 0.81, 95% CI: 0.10 to 1.53, *p* = 0.026). IL-10 showed a non-significant increase (SMD = 0.66, 95% CI: −0.09 to 1.41, *p* = 0.084), with no effect on IFN-γ. Heterogeneity was moderate for CRP (I^2^ = 52.5%) but high for other markers (I^2^ > 87%). These findings suggest that exerkines contribute to an anti-inflammatory shift in the short term, which is consistent with mechanisms that may underlie the preventive effects of exercise against cardiometabolic diseases; however, standardized protocols and longitudinal studies with clinical endpoints are needed to confirm any long-term benefits.

## 1. Introduction

Physical exercise is widely recognized as a cornerstone of health promotion and disease prevention, exerting its benefits through the complex interplay of physiological mechanisms [1]. Among these, the release of exercise-induced cytokines, often termed “exerkines,” has emerged as a critical mediator linking acute physical activity to systemic health outcomes [2]. Exerkines, including interleukin-6 (IL-6), tumor necrosis factor-alpha (TNF-α), and interleukin-10 (IL-10), are secreted by skeletal muscle and other tissues during exercise, modulating inflammatory responses, metabolism, and tissue repair [3]. Although these cytokines typically peak within hours of exercise and return to baseline soon after, growing evidence suggests that such transient responses may trigger cumulative adaptations, potentially offering long-term protection against chronic diseases such as cardiovascular disease, type 2 diabetes, and obesity [4,5]. Additionally, C-reactive protein (CRP), a hepatic acute-phase protein modulated by exerkines like IL-6, and Interferon-γ (IFN-γ), a cytokine with immunomodulatory properties, also contribute to the regulation of the inflammatory state, as explored in subsequent sections [6].

The mechanisms underlying these long-term effects are multifaceted. Notably, transient exerkine elevations may drive prolonged benefits by modulating immune responses, a process central to their protective role. Repeated exercise-induced signaling, mediated by pathways such as nuclear factor-kappa B (NF-κB) and Janus kinase/signal transducer and activator of transcription (JAK/STAT), can shift the immune milieu toward an anti-inflammatory state, reducing chronic low-grade inflammation—a known driver of metabolic and cardiovascular disorders [4,7,8]. For instance, IL-6, often elevated acutely during exercise, promotes the production of anti-inflammatory cytokines, such as IL-10, while suppressing pro-inflammatory mediators like TNF-α, thereby potentially fostering a balanced immune profile over time [9]. Beyond immunity, these adaptations may support long-term cardiometabolic health through anti-inflammatory pathways [4,10]. Additionally, epigenetic modifications, such as DNA methylation changes in immune and metabolic genes, and improved mitochondrial biogenesis—driven by factors like peroxisome proliferator-activated receptor gamma coactivator 1-alpha (PGC-1α)—may amplify cellular resilience, may collectively contribute to reduced disease risk [5,11,12]. These converging mechanisms highlight the possibility that short-lived exerkines could leave a lasting imprint on health, prompting the need for a systematic synthesis of the evidence.

Despite compelling evidence linking exerkines to acute physiological changes, significant gaps remain in our understanding of their broader implications. Most research has examined the immediate effects in trained athletes or clinical populations, leaving the impact on healthy adults and those with or at risk for chronic conditions largely unexplored [13,14]. This focus has left open questions about the consistency of exerkine responses and their long-term relevance. For instance, while some studies report robust cytokine shifts following exercise, the factors driving these responses—such as exercise modality, intensity, or individual characteristics—remain inconsistently characterized across the literature [15,16]. Moreover, although systematic reviews have explored myokines in the context of disease management or broader metabolic effects, few have specifically addressed the capacity of short-lived exerkines to contribute to long-term health outcomes in healthy individuals [17]. This scarcity of synthesized evidence is particularly notable given the emerging interest in exerkines as potential biomarkers and therapeutic targets. For example, IL-6, traditionally viewed as a pro-inflammatory cytokine, exhibits context-dependent anti-inflammatory properties during exercise, suggesting a complex role that requires further clarification [18]. As chronic diseases continue to impose a substantial burden on global health systems, understanding whether these transient signals can translate into sustained protective effects is critical for advancing preventive strategies [19]. Thus, a systematic review and meta-analysis are needed to consolidate the evidence, address these unresolved questions, and elucidate the long-term preventive potential of exerkines in healthy populations. We therefore quantitatively synthesized evidence from RCTs on short-term changes in key exerkines and inflammatory markers to clarify their immunomodulatory role.

The primary aim of this systematic review and meta-analysis was to quantify the effects of exercise interventions on key exerkines (IL-6, TNF-α, IL-10) and related inflammatory markers (CRP, IFN-γ) in randomized controlled trials involving healthy individuals and those with or at risk of chronic conditions. We focused on pre- to post-intervention changes to assess the immunomodulatory role of short-lived exerkines. These biomarker shifts were interpreted in the context of established evidence linking regular exercise to reduced chronic disease risk, with the recognition that direct evidence of long-term prevention requires studies with clinical endpoints.

## 2. Materials and Methods

### 2.1. Design

A systematic review was conducted following the Preferred Reporting Items for Systematic Reviews and Meta-Analyses (PRISMA) 2020 statement [20]. The systematic review protocol was pre-registered in PROSPERO with the identification number CRD42025649283.

### 2.2. Search Strategy

Two independent researchers retrieved articles from PubMed (MEDLINE), Web of Science, and Scopus as of 7 February 2025. The keywords (“exercise” OR “physical activity” OR “aerobic exercise” OR “resistance training” OR “high-intensity interval training” OR “exercise training”) AND (“cytokine” OR “cytokines” OR “pro-inflammatory cytokines” OR “anti-inflammatory cytokines” OR “exerkine” OR “exerkines”) AND (“disease prevention” OR “chronic disease” OR “long-term health” OR “health outcomes”) were used to conduct the searches. The literature search parameters were designed to include all English-language articles from January 2015 to February 2025, excluding reviews and meta-analyses. An updated search conducted up to 1 July 2025 identified no additional eligible studies. All steps in the record screening process were managed with EndNote. In addition, the references of all collected papers were reviewed to discover additional related studies.

### 2.3. Eligibility Criteria

The eligibility criteria were defined according to the PICOS framework. For the population, studies included adults aged 18 years or older, comprising healthy individuals, those diagnosed with specific chronic conditions (e.g., sarcopenia, rheumatoid arthritis, cognitive impairment), or those at elevated risk of chronic diseases (e.g., individuals with heightened breast cancer susceptibility). Interventions consisted of structured exercise regimens, including aerobic, resistance, combined, or high-intensity interval training, characterized by specified duration and intensity, without concurrent dietary or pharmacological co-interventions. Comparison groups consisted of non-exercising or inactive controls who maintained their habitual lifestyle without structured interventions. Outcomes encompassed primary measures of short-term alterations in circulating exerkine concentrations (e.g., IL-6, TNF-alpha, IL-10, CRP, IFN-gamma) assessed pre- and post-intervention, and secondary endpoints related to chronic disease prevention or management, including systemic inflammation, metabolic parameters, and cardiovascular performance. Study designs were restricted to randomized controlled trials (RCTs) featuring a control arm, published in English in peer-reviewed journals.

### 2.4. Exclusion Criteria

Studies were excluded if they involved animal models or in vitro experiments, lacked a clearly defined control group, or included confounding interventions (e.g., dietary or pharmacological treatments). Non-peer-reviewed publications, such as conference abstracts, reviews, or letters, were ineligible, as were duplicate studies, those with irretrievable data despite author contact, or studies published before January 2015 or in languages other than English.

### 2.5. Data Extraction

A customized data extraction template was developed and piloted in Microsoft Excel to systematically capture pertinent information from the included studies. Data extraction was conducted independently by the primary reviewer (H.P.), with the following variables recorded: (1) study identification (first author, publication year, country); (2) study design (e.g., RCT, parallel or crossover); (3) participant characteristics (sample size, age, sex, health status); (4) intervention details (exercise type, duration, frequency, intensity, and mode of delivery); (5) comparator details (non-exercising or inactive control group); (6) outcome measures, including primary outcomes (pre- and post-intervention concentrations of circulating exerkines such as IL-6, TNF-alpha, IL-10, CRP, IFN-gamma) and secondary outcomes (systemic inflammation markers, metabolic parameters, cardiovascular performance, and adverse events); and (7) key findings. Quantitative data, including means, standard deviations (SDs), and sample sizes for intervention and control groups at baseline and post-intervention, were extracted to enable meta-analytic calculations. For studies presenting data solely in graphical format, numerical values were extracted with high precision using Web Plot Digitizer software (version 4.6, Automeris LLC, 2023) [21]. In instances where critical data were missing or unclear, corresponding authors were contacted via email, allowing three days for response. Studies with unresolved data gaps were excluded from the quantitative synthesis for the affected outcomes. All extracted data were cross-verified by the second reviewer (R.E.), with discrepancies resolved through consensus or consultation with a third reviewer.

### 2.6. Statistical Analysis

Meta-analyses were performed using Comprehensive Meta-Analysis software (version 3, Biostat, Inc., Englewood, NJ, USA). Standardized mean differences (SMDs) with 95% confidence intervals (CIs) were calculated to evaluate the effects of exercise interventions on circulating exerkine levels, including IL-6, TNF-alpha, IL-10, CRP, and IFN-gamma. A random-effects model was applied to account for heterogeneity due to variations in exercise protocols and participant characteristics. Heterogeneity was assessed using the I^2^ statistic (I^2^ > 50%: moderate; I^2^ > 75%: high). Subgroup analyses were planned a priori to explore sources of heterogeneity where sufficient data were available (≥2 studies per subgroup), including exercise modality (aerobic vs. resistance vs. combined/others), health status (healthy vs. chronic condition/at-risk), and intervention duration (<12 weeks vs. ≥12 weeks). Due to the limited number of studies per outcome and subgroup (e.g., only 2 aerobic, 4 resistance), formal subgroup analyses and meta-regression could not be conducted. This limitation is acknowledged in the discussion. Sensitivity analyses used the “one study removed” approach. Publication bias was evaluated with funnel plots and Egger’s test (*p* < 0.05 indicating potential bias). Trim and Fill analysis adjusted for potential bias across all outcomes. Statistical significance was set at *p* < 0.05 (two-sided).

## 3. Results

### 3.1. Study Selection

**Figure 1:**
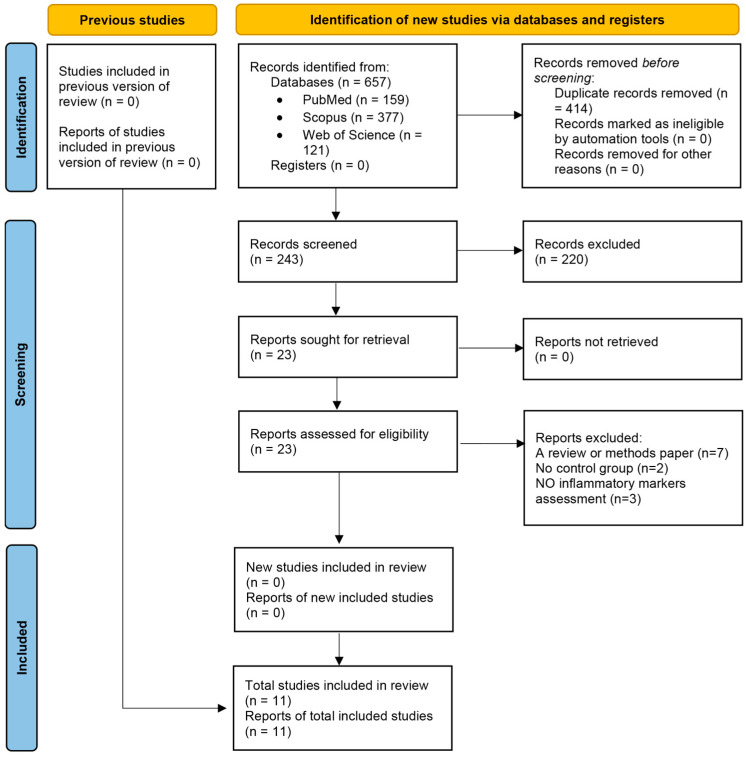
Flow diagram regarding article selection for the meta-analysis.

A systematic search of PubMed (MEDLINE), Scopus, and Web of Science up to 7 February 2025 identified 657 records (PubMed: 159, Scopus: 377, Web of Science: 121). After removing 414 duplicates using EndNote’s de-duplication feature, 243 unique records remained. Title and abstract screening excluded 220 records, leaving 23 reports for full-text review. Of these, 23 reports were assessed for eligibility, with 12 excluded (7 reviews or methods papers, 2 without a control group, 3 with no inflammatory markers assessment), resulting in 11 randomized controlled trials (RCTs) involving 1135 participants were included in the meta-analysis. The PRISMA 2020 flow diagram (Figure 1) illustrates the selection process, with detailed reasons for exclusion provided in Supplementary Table S1.

### 3.2. Study Characteristics

The 11 randomized controlled trials, published between 2016 and 2022, were conducted in North America (*n* = 2), South America (*n* = 1), Europe (*n* = 5), and Asia (*n* = 3). Participants included healthy postmenopausal women (*n* = 2 studies), individuals with chronic conditions such as rheumatoid arthritis, cognitive impairment, or sarcopenia (*n* = 6), and those at elevated risk of chronic diseases, including obesity, metabolic syndrome, or high breast cancer risk (*n* = 3). The total sample size was 1135 participants (1054 females, 81 males), with individual study sizes ranging from 24 to 720 participants. Exercise interventions consisted of aerobic training (*n* = 2), resistance training (*n* = 4), combined aerobic and resistance training (n = 3), and high-intensity interval training (*n* = 2), with durations ranging from 6 to 52 weeks and frequencies of 2 to 5 sessions per week. Control groups maintained their habitual lifestyle without structured exercise. Primary outcomes focused on circulating exerkines, including IL-6, TNF-α, IL-10, CRP, and IFN-γ. Secondary outcomes included body composition, cardiovascular fitness, and metabolic parameters. Detailed study characteristics are presented in Supplementary Table S2.

### 3.3. Risk of Bias in Included Studies

**Figure 2:**
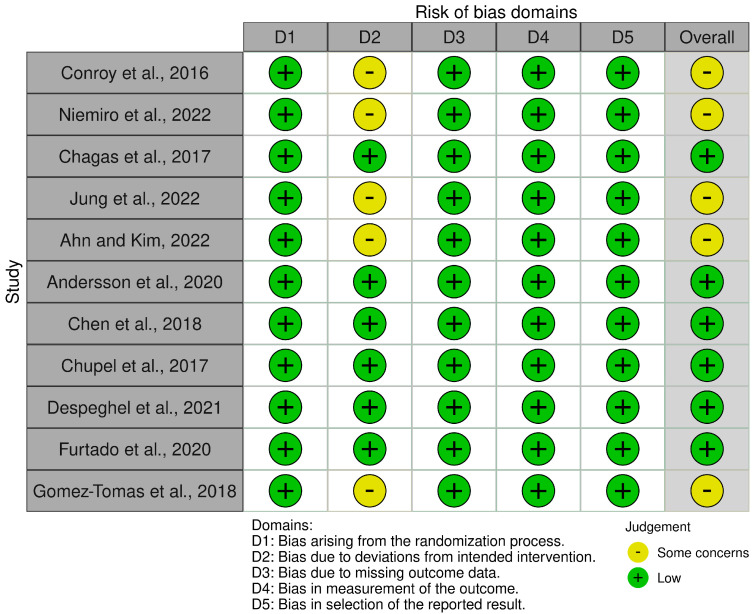
Summary of risk of bias assessments for the 11 included randomized controlled trials using the Cochrane Risk of Bias 2 (RoB 2) tool [22,23,24,25,26,27,28,29,30,31,32].

Risk of bias for the 11 included RCTs was assessed using the Cochrane Risk of Bias 2 (RoB 2) tool, evaluating five domains: randomization process (D1), deviations from intended interventions (D2), missing outcome data (D3), measurement of the outcome (D4), and selection of the reported result (D5). Two reviewers (H.P. and R.E.) conducted assessments independently, with disagreements resolved by a third reviewer (B.K.). Overall, six studies [22,23,24,25,26,27] were rated as having a low risk of bias across all domains. Five studies [28,29,30,31,32] raised some concerns, primarily due to potential bias in D2 (deviations from intended interventions) related to the lack of blinding of participants and personnel. No studies were rated as having a high overall risk of bias. Detailed results are presented in Supplementary Table S3 and summarized in Figure 2.

### 3.4. Meta-Analysis Results

#### 3.4.1. Effect of Exercise on C-Reactive Protein (CRP) Levels

**Figure 3:**
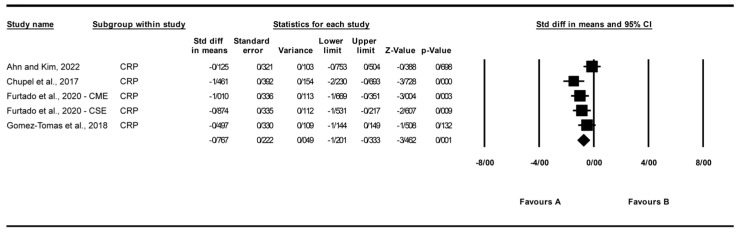
Meta-analysis of the effects of exercise interventions on CRP [25,27,31,32]. Each black square represents the standardized mean difference (SMD) for an individual study, with the size of the square proportional to the study weight in the random-effects model. Horizontal lines indicate 95% confidence intervals (CIs). The diamond represents the pooled SMD with its 95% CI.

A total of five studies were analyzed to assess the impact of exercise interventions on the inflammatory marker C-reactive protein (CRP), a hepatic acute-phase protein modulated by exerkines such as IL-6, reflecting the study’s focus on exercise effects on inflammatory markers [25,27,31,32]. The findings indicated that exercise significantly reduced CRP levels (standardized mean difference [SMD] [24] c = −0.77, 95% CI: −1.20 to −0.33, *p* = 0.001; Figure 3) compared to the control group. The studies exhibited moderate and non-significant heterogeneity (I^2^ = 52.5%, *p* = 0.077). Visual analysis of the funnel plot (Supplementary Figure S1) revealed moderate asymmetry, with most studies favoring a reduction in CRP levels, though Egger’s test indicated no significant publication bias (*p* = 0.072). Trim and Fill analysis under a random-effects model identified no missing studies, yielding an adjusted SMD of −0.77 (95% CI: −1.20 to −0.33), consistent with the original effect, suggesting stability despite observed asymmetry. Sensitivity analysis using the “one study removed” approach confirmed the significant reduction in C-reactive protein (CRP) levels with exercise (SMD = −0.767, 95% CI: −1.201 to −0.333, *p* = 0.001). Excluding [31] yielded a larger effect (SMD = −0.927, 95% CI: −1.301 to −0.553, *p* < 0.001). Other exclusions produced SMDs from −0.618 to −0.843 (all *p* < 0.01), indicating no single study drove the effect (Supplementary Figure S6).

#### 3.4.2. Effect of Exercise on Interferon-Gamma (IFN-γ) Levels

**Figure 4:**
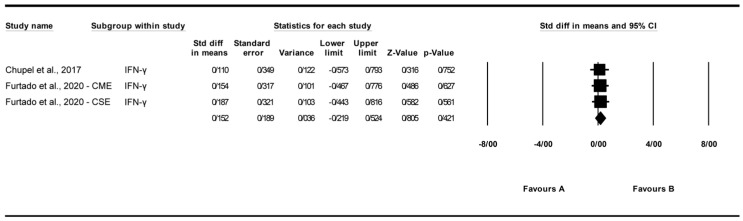
Meta-analysis of the effects of exercise interventions on IFN-γ [25,27]. Each black square represents the standardized mean difference (SMD) for an individual study, with the size of the square proportional to the study weight in the random-effects model. Horizontal lines indicate 95% confidence intervals (CIs). The diamond represents the pooled SMD with its 95% CI.

A total of three studies were analyzed to assess the impact of exercise interventions on interferon-gamma (IFN-γ) levels [25,27]. The findings indicated that exercise had no significant effect on IFN-γ levels (standardized mean difference [SMD] = 0.152, 95% CI: −0.219 to 0.524, *p* = 0.421; Figure 4) compared to the control group. The studies exhibited no significant heterogeneity (I^2^ = 0%, *p* = 0.987). Funnel plot analysis (Supplementary Figure S2) showed relative symmetry, supported by Egger’s test (*p* = 0.374) and Begg’s test (*p* = 0.602), indicating no significant publication bias. Trim and Fill analysis under a random-effects model imputed no additional studies (observed SMD = 0.152, 95% CI: −0.219 to 0.524; adjusted SMD = 0.152, 95% CI: −0.219 to 0.524), confirming the stability of the effect. Sensitivity analysis using the “one study removed” approach confirmed the non-significant effect of exercise on interferon-gamma (IFN-γ) levels (SMD = 0.152, 95% CI: −0.219 to 0.524, *p* = 0.421). Excluding [25] yielded an SMD of 0.170 (95% CI: −0.272 to 0.612, *p* = 0.451). Other exclusions produced SMDs of 0.134 to 0.152 (*p* > 0.4), indicating no single study drove the effect (Supplementary Figure S7).

#### 3.4.3. Effect of Exercise on Interleukin-6 (IL-6) Levels

**Figure 5:**
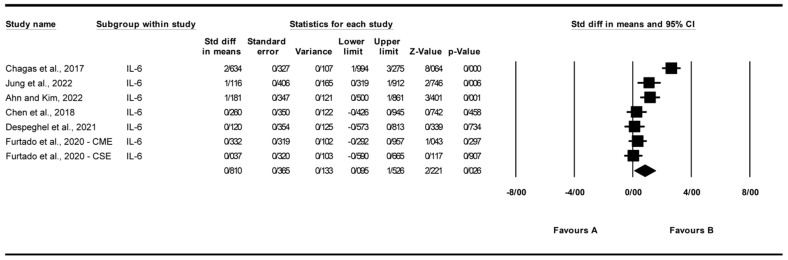
Meta-analysis of the effects of exercise interventions on IL-6 [22,24,26,27,30,31]. Each black square represents the standardized mean difference (SMD) for an individual study, with the size of the square proportional to the study weight in the random-effects model. Horizontal lines indicate 95% confidence intervals (CIs). The diamond represents the pooled SMD with its 95% CI.

A total of seven studies were analyzed to assess the impact of exercise interventions on interleukin-6 (IL-6) levels, with [27] contributing two subgroups: chair elastic band muscle-strength exercise (CSE) and chair multimodal exercise (CME) [22,24,26,27,30,31]. The findings indicated that exercise significantly affected IL-6 levels (standardized mean difference [SMD] = 0.810, 95% CI: 0.095 to 1.526, *p* = 0.026; Figure 5) compared to the control group. The studies exhibited high and significant heterogeneity (I^2^ = 87.298%, *p* = 0.000). Funnel plot analysis (Supplementary Figure S3) showed relative symmetry, supported by Egger’s test (*p* = 0.944) and Begg’s test (*p* = 0.881), indicating no significant publication bias. Trim and Fill analysis under a random-effects model imputed no additional studies (observed SMD = 0.494, 95% CI: 0.272 to 1.612; adjusted SMD = 0.494, 95% CI: 0.272 to 1.612), suggesting stability of the effect despite a Q value of 47.235 indicating some variability across studies. Sensitivity analysis using the “one study removed” approach confirmed the robustness of the effect, with SMD ranging from 0.485 (95% CI: 0.090 to 0.879, *p* = 0.016) after removing [22] to 0.942 (95% CI: 0.150 to 1.734, *p* = 0.020) after removing [27]. All *p*-values remained significant (*p* < 0.05) except when removing [30] (*p* = 0.068) or [31] (*p* = 0.078), indicating that the overall effect is not driven by a single study (Supplementary Figure S8). The significant effect on IL-6 was lost upon removal of [30] (*p* = 0.068) or [31] (*p* = 0.078), indicating fragility of the pooled estimate.

#### 3.4.4. Effect of Exercise on Interleukin-10 (IL-10) Levels

**Figure 6:**
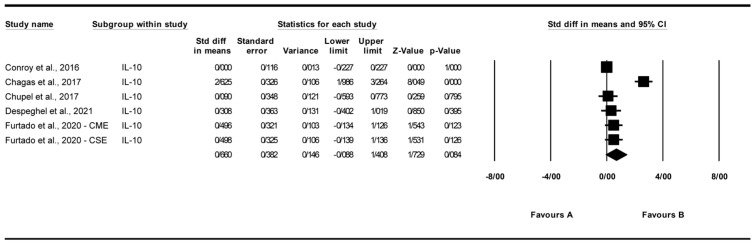
Meta-analysis of the effects of exercise interventions on IL-10 [22,25,26,27,28]. Each black square represents the standardized mean difference (SMD) for an individual study, with the size of the square proportional to the study weight in the random-effects model. Horizontal lines indicate 95% confidence intervals (CIs). The diamond represents the pooled SMD with its 95% CI.

A total of six studies were analyzed to assess the impact of exercise interventions on interleukin-10 (IL-10) levels [22,25,26,27,28]. The findings indicated a non-significant trend toward increased IL-10 levels with exercise (standardized mean difference [SMD] = 0.660, 95% CI: −0.088 to 1.408, *p* = 0.084; Figure 6) compared to the control group. The studies exhibited high and significant heterogeneity (I^2^ = 91.466%, *p* = 0.000). Visual analysis of the funnel plot (Supplementary Figure S4) revealed slight asymmetry, though Egger’s test indicated no significant publication bias (*p* = 0.262). Trim and Fill analysis under a random-effects model imputed two studies, yielding an adjusted SMD of 0.699 (95% CI: 0.176 to 1.794), compared to the original SMD of 0.660 (95% CI: −0.088 to 1.408, *p* = 0.084). Sensitivity analysis using the “one study removed” approach showed that the non-significant trend in IL-10 levels (SMD = 0.660, 95% CI: −0.088 to 1.408, *p* = 0.084) was influenced by [22]. Excluding this study reduced the SMD to 0.121 (95% CI: −0.072 to 0.314, *p* = 0.220). Other exclusions produced SMDs from 0.694 to 0.808 (*p* > 0.08), indicating no single study altered the overall non-significant effect (Supplementary Figure S9).

#### 3.4.5. Effect of Exercise on Tumor Necrosis Factor-Alpha (TNF-α) Levels

**Figure 7:**
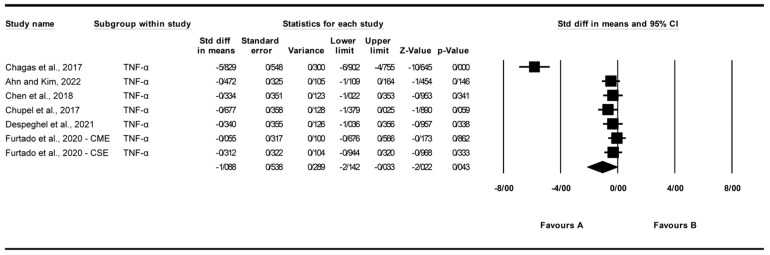
Meta-analysis of the effects of exercise interventions on TNF-α [22,24,25,26,27,31]. Each black square represents the standardized mean difference (SMD) for an individual study, with the size of the square proportional to the study weight in the random-effects model. Horizontal lines indicate 95% confidence intervals (CIs). The diamond represents the pooled SMD with its 95% CI.

A total of seven studies were analyzed to assess the impact of exercise interventions on tumor necrosis factor-alpha (TNF-α) levels [22,24,25,26,27,31]. The findings indicated that exercise significantly reduced TNF-α levels (standardized mean difference [SMD] = −1.088, 95% CI: −2.142 to −0.033, *p* = 0.043; Figure 7) compared to the control group. The studies exhibited high and significant heterogeneity (I^2^ = 93.744%, *p* = 0.000). Funnel plot analysis (Supplementary Figure S5) indicated asymmetry, supported by Egger’s test (*p* = 0.00038) and Begg’s test (*p* = 0.011), suggesting significant publication bias. Trim and Fill analysis under a random-effects model imputed two additional studies (observed SMD = −1.088, 95% CI: −2.142 to −0.033; adjusted SMD = −1.222, 95% CI: −1.474 to −0.490), indicating a potential overestimation of the effect due to missing studies on the left of the mean, which shifted the effect further toward a negative direction. Sensitivity analysis using the “one study removed” approach revealed that the significant reduction in TNF-α levels was partially driven by [22]. When this study was excluded, the effect size was reduced but remained significant (SMD = −0.354, 95% CI: −0.623 to −0.084, *p* = 0.010; Supplementary Figure S10). The significant reduction in TNF-α was heavily influenced by [22]; exclusion reduced the effect but retained significance (SMD = −0.35, *p* = 0.010), highlighting limited robustness.

## 4. Discussion

This meta-analysis of 11 RCTs demonstrates short-term immunomodulatory effects of exercise, with significant reductions in pro-inflammatory CRP (SMD = −0.77, 95% CI: −1.20 to −0.33, *p* = 0.001) and TNF-α (SMD = −1.088, 95% CI: −2.142 to −0.033, *p* = 0.043; I^2^ = 93.7%), alongside increased IL-6 (SMD = 0.810, 95% CI: 0.095 to 1.526, *p* = 0.026; I^2^ = 87.3%) with sensitivity to individual studies, and a non-significant trend for IL-10 (SMD = 0.660, 95% CI: −0.088 to 1.408, *p* = 0.084). These changes reflect acute modulation of the systemic inflammatory milieu in adults, including healthy individuals and those with or at risk of chronic conditions. Conducted per PRISMA 2020 guidelines [20]. the analysis synthesized evidence on exercise-induced exerkines (CRP, IL-6, IL-10, TNF-α, IFN-γ) and their potential mechanistic role in cardiometabolic health [4,19].

The reductions in CRP and TNF-α are clinically significant, given their established associations with cardiometabolic disorders, including cardiovascular disease, type 2 diabetes, and obesity [10,33]. Sustained reductions in CRP have been linked to a lower incidence of cardiovascular events in longitudinal studies, suggesting that the observed changes may serve as intermediate biomarkers for long-term risk reduction [33]. Decreased TNF-α levels may enhance insulin sensitivity and mitigate atherogenesis, thereby suggesting a potential contribution to cardiometabolic health [7]. The significant increase in IL-6, often misconstrued as solely pro-inflammatory, reflects its context-dependent anti-inflammatory role during exercise, where it stimulates IL-10 production and suppresses TNF-α [2,9]. This anti-inflammatory effect of IL-6 is mediated through key signaling pathways, including nuclear factor-kappa B (NF-κB) and Janus kinase/signal transducer and activator of transcription (JAK/STAT). During exercise, IL-6 inhibits the pro-inflammatory NF-κB pathway in immune cells, reducing the production of TNF-α and other inflammatory mediators [2,34]. Concurrently, IL-6 activates the JAK/STAT pathway, particularly STAT3, which promotes the transcription of anti-inflammatory genes such as IL-10, thereby fostering an anti-inflammatory milieu [9,35]. These molecular mechanisms provide a plausible link between transient exerkine responses and sustained physiological adaptations that may mitigate chronic disease risk. The trend toward elevated IL-10 supports a shift toward immune homeostasis, counteracting pro-inflammatory mediators [36]. These findings align with the hypothesis that transient exerkine responses may trigger cumulative physiological adaptations with protective effects against chronic diseases [4,19].

Exercise-induced exerkines may contribute to long-term health through additional mechanisms beyond inflammation. IL-6 has been shown to upregulate brain-derived neurotrophic factor (BDNF), promoting neuroplasticity and potentially reducing the risk of cognitive decline [37]. Epigenetic modifications, such as DNA methylation of genes involved in inflammation and metabolism, have been associated with regular exercise and may persist in altering cardiometabolic risk profiles [12,38]. Enhanced mitochondrial biogenesis, mediated by peroxisome proliferator-activated receptor gamma coactivator 1-alpha (PGC-1α), improves cellular energy metabolism and resilience, could support long-term cardiometabolic health [39]. Resistance exercise may uniquely modulate immunoregulatory myokines, potentially explaining variability in exerkine responses across modalities [34].

Substantial heterogeneity in IL-6 (I^2^ = 87.3%) and TNF-α (I^2^ = 93.7%) outcomes poses a critical challenge to interpretation. Variability likely arises from differences in exercise intensity, timing of blood sampling relative to sessions, and assay sensitivity across trials. Sensitivity analyses underscore this instability: the IL-6 effect lost significance upon exclusion of [30] or [31], while TNF-α was disproportionately influenced by [22]. Thus, these findings must be viewed cautiously until replicated in more uniform study designs.

The study population was overwhelmingly female (93%; 1054 females vs. 81 males), markedly limiting applicability to males. Sex-specific hormonal profiles, including testosterone and estrogen fluctuations, may differentially regulate exerkine secretion and downstream inflammatory pathways in men [40]. Consequently, the observed effects are primarily relevant to females, and dedicated trials in male cohorts are required to establish generalizability.

The findings of this meta-analysis highlight the immunomodulatory potential of exercise, although several methodological limitations warrant careful consideration. The high heterogeneity observed for IL-6 (I^2^ = 87.3%), IL-10 (I^2^ = 91.5%), and TNF-α (I^2^ = 93.7%) represents a major limitation, precluding definitive conclusions and limiting the robustness of pooled estimates. Although subgroup analyses were planned a priori to explore sources of heterogeneity—including exercise modality, intervention duration, and participant health status—the limited number of studies per subgroup rendered them infeasible. Similarly, meta-regression was not possible due to the small overall study pool (*n* = 11). Sensitivity analyses further revealed the fragility of the pooled effects, with the IL-6 result becoming non-significant upon removal of [30] or [31], and the TNF-α effect was heavily influenced by [22]. Future meta-analyses with larger, more homogeneous study sets should prioritize subgroup and meta-regression analyses to resolve these sources of variability. Moderate heterogeneity for CRP (I^2^ = 52.5%) and potential publication bias for TNF-α (Egger’s test, *p* = 0.00038) further influence the precision of effect estimates, while sample sizes ranging from 112 to 522 participants and variability in exercise protocols or participant demographics, as seen in studies like [22], add to this complexity. The exclusion of additional exerkines such as hs-CRP and IL-4 due to limited data [25,28] also narrows the analysis scope. Given the short-term nature of the interventions, longitudinal studies with clinical endpoints such as cardiovascular disease or diabetes incidence are essential to confirm long-term benefits [19]. A key limitation of this analysis is that it is restricted to short-term changes in inflammatory markers (typically assessed within 6–52 weeks of intervention) and does not include long-term clinical outcomes such as disease incidence or mortality. While reductions in CRP and TNF-α, alongside elevations in IL-6, are consistent with an anti-inflammatory adaptation known to accompany regular exercise, any direct causal link to chronic disease prevention remains hypothetical. Longitudinal cohort studies with hard clinical endpoints are required to establish whether these transient exerkine responses translate into sustained risk reduction. Despite these considerations, the consistent reductions in CRP and TNF-α and the increase in IL-6 highlight the promising role of exercise in modulating systemic inflammation.

Future research must address current limitations through rigorously designed investigations. Standardized exercise protocols that specify modality, intensity, duration, frequency, and the precise timing of blood sampling relative to sessions are critical to minimize heterogeneity and enhance comparability across studies. Longitudinal RCTs with hard clinical endpoints such as incident cardiovascular events, type 2 diabetes diagnosis, or all-cause mortality and follow-up durations of at least 1 year are essential to determine whether transient exerkine shifts confer sustained preventive benefits. Balanced sex representation targeting at least 40% male participants is imperative to correct the existing female-dominant bias and delineate potential sex-specific exerkine dynamics. Finally, integrating multi-omics profiling, such as transcriptomics and metabolomics, with dose-response modeling will elucidate mechanistic pathways and support personalized exercise prescriptions.

## 5. Conclusions

This meta-analysis of 11 RCTs shows that exercise induces short-term reductions in CRP and TNF-α and elevations in IL-6, consistent with an anti-inflammatory shift in the inflammatory profile. These transient exerkine-mediated changes may underpin the long-term benefits of regular exercise against cardiometabolic and inflammatory diseases. However, the lack of long-term clinical endpoints in the included studies precludes firm conclusions on disease prevention. Longitudinal trials with hard clinical outcomes are essential to confirm whether these biomarker shifts translate into sustained risk reduction. Future research should prioritize standardized protocols, balanced sex representation, and clinical endpoint assessment to establish the translational relevance of these findings.

## Data Availability

The data analyzed in this study are derived from publicly available studies included in the meta-analysis and are referenced accordingly in the manuscript.

## Supplementary Materials

The following supporting information can be downloaded at: https://www.mdpi.com/article/10.3390/biom15111590/s1, Tables S1–S3: Search strategy, study characteristics, and risk of bias assessments; Figures S1–S10: Funnel plots and sensitivity analyses. Table S1: Comprehensive search strategy across PubMed (MEDLINE), Scopus, and Web of Science databases, including search strings, filters, dates, retrieved records, and screening outcomes. An updated search up to 1 July 2025 identified no additional eligible studies. Table S2: Detailed characteristics and outcomes of the 11 included randomized controlled trials, including study design, sample size (intervention/control, gender), population characteristics (health status, country), intervention specifics (type, duration, frequency, intensity), primary outcomes (circulating exerkine levels: IL-6, TNF-α, IL-10, CRP, IFN-γ), secondary outcomes (e.g., body composition, cardiovascular fitness, metabolic parameters), key results, and risk of bias assessments using the Cochrane Risk of Bias 2 (RoB 2) tool. Table S3: Risk of bias assessments for the 11 included randomized controlled trials using the Cochrane Risk of Bias 2 (RoB 2) tool. The table evaluates five domains: randomization process, deviations from intended interventions, missing outcome data, measurement of the outcome, and selection of the reported result. Each domain is rated as low risk, some concerns, or high risk, with an overall risk of bias determined for each study. Figure S1: Funnel plot for C-reactive protein (CRP). The plot depicts standardized mean difference (SMD) and standard error (SE) for studies assessing the effect of exercise interventions on CRP levels. The plot includes data from five studies [25,27,31,32]. Vertical line represents the pooled SMD (−0.77, 95% CI: −1.20 to −0.33), with dashed lines indicating the 95% confidence interval. Figure S2: Funnel plot for interferon-gamma (IFN-γ). The plot depicts SMD and SE for studies assessing the effect of exercise interventions on IFN-γ levels. The plot includes data from three studies [25,27]. Vertical line represents the pooled SMD (0.15, 95% CI: −0.22 to 0.52), with dashed lines indicating the 95% confidence interval. Figure S3: Funnel plot for interleukin-6 (IL-6). The plot depicts SMD and SE for studies assessing the effect of exercise interventions on IL-6 levels. The plot includes data from seven studies [22,26,27,30,31]. Vertical line represents the pooled SMD (0.81, 95% CI: 0.10 to 1.53), with dashed lines indicating the 95% confidence interval. Figure S4: Funnel plot for interleukin-10 (IL-10). The plot depicts SMD and SE for studies assessing the effect of exercise interventions on IL-10 levels. The plot includes data from six studies [22,25,26,27,28]. Vertical line represents the pooled SMD (0.66, 95% CI: −0.09 to 1.41), with dashed lines indicating the 95% confidence interval. Figure S5: Funnel plot for tumor necrosis factor-alpha (TNF-α). The plot depicts SMD and SE for studies assessing the effect of exercise interventions on TNF-α levels. The plot includes data from seven studies [22,24,25,26,27,31]. Vertical line represents the pooled SMD (−1.09, 95% CI: −2.14 to −0.03), with dashed lines indicating the 95% confidence interval. Figure S6: Sensitivity analysis for C-reactive protein (CRP) using the “one study removed” method. The plot shows SMD and SE for CRP with one study removed. Pooled SMD = −0.767 (95% CI: −1.201 to −0.333), SMDs from −0.618 to −0.927 (all *p* < 0.01). Figure S7: Sensitivity analysis for interferon-gamma (IFN-γ) using the “one study removed” method. The plot shows pooled SMD and SE for IFN-γ with one study removed. Pooled SMD = 0.152 (95% CI: −0.219 to 0.524), with no significant effect (*p* = 0.421). Figure S8: Sensitivity analysis for interleukin-6 (IL-6) using the “one study removed” method. The plot shows SMD and SE for IL-6 with one study removed. Pooled SMD = 0.810 (95% CI: 0.095 to 1.526), with SMDs ranging from 0.485 to 0.942 (all *p* < 0.05, except specific exclusions). Figure S9: Sensitivity Analysis for Interleukin-10 (IL-10) using the “one study removed” method. The plot shows SMD and SE for IL-10 with one study removed. Pooled SMD = 0.660 (95% CI: −0.088 to 1.408), with SMDs ranging from 0.121 to 0.808 (*p* > 0.08). Figure S10: Sensitivity Analysis for Tumor Necrosis Factor-Alpha (TNF-α) using the “one study removed” method. The plot shows SMD and SE for TNF-α with one study removed. Pooled SMD = −1.088 (95% CI: −2.142 to −0.033), with SMDs ranging from −0.354 to −1.222 (*p* < 0.05, except specific exclusions).

## Abbreviations

The following abbreviations are used in this manuscript:

IL-6	Interleukin-6
TNF-alpha	Tumor Necrosis Factor-alpha
IL-10	Interleukin-10
CRP	C-reactive protein
IFN- γ	Interferon-gamma
SMD	Standardized Mean Difference
CI	Confidence Interval
RCT	Randomized Controlled Trial
NF-kappaB	Nuclear Factor-kappa B
JAK/STAT	Janus Kinase/Signal Transducer and Activator of Transcription
PGC-1alpha	Peroxisome Proliferator-Activated Receptor Gamma Coactivator 1-alpha
