# Supplementary material for: Short-Lived Exercise-Induced Exerkines Modulate Inflammation for Chronic Disease Prevention: A Systematic Review and Meta-Analysis
Source: Biomolecules. 2025 Nov 13;15(11):1590. doi: 10.3390/biom15111590 (PMC12650156; doi:10.3390/biom15111590)
Supplement: Supplementary file 1 [file biomolecules-15-01590-s001.zip › biomolecules-3859678-supplementary.pdf]

Table S1. Comprehensive Search Strategy.

Database	Search String	Filters Applied	Date of Search	Records Retrieved	Screening Outcomes
PubMed (MEDLINE)	("cytokine" OR "cytokines" OR "pro-inflammatory cytokines" OR "anti-inflammatory cytokines" OR "exerkine" OR "exerkines") AND ("exercise" OR "physical activity" OR "aerobic exercise" OR "resistance training" OR "high-intensity interval training" OR "exercise training") AND ("disease prevention" OR "chronic disease" OR "long-term health" OR "health outcomes") NOT (review[pt] OR meta-analysis[pt])	English language, January 2015–February 2025, original research articles	7-Feb-25	159	105 English-language records; 80 after title/abstract screening
Scopus	TITLE-ABS-KEY(("cytokine" OR "cytokines" OR "pro-inflammatory cytokines" OR "anti-inflammatory cytokines" OR "exerkine" OR "exerkines") AND ("exercise" OR "physical activity" OR "aerobic exercise" OR "resistance training" OR "high-intensity interval training" OR "exercise training") AND ("disease prevention" OR "chronic disease" OR "long-term health" OR "health outcomes"))	English language, January 2015–February 2025, original research articles	7-Feb-25	377	165 English-language records; 120 after title/abstract screening
Web of Science	TS=("cytokine" OR "cytokines" OR "pro-inflammatory cytokines" OR "anti-inflammatory cytokines" OR "exerkine" OR "exerkines") AND TS=("exercise" OR "physical activity" OR "aerobic exercise" OR "resistance training" OR "high-intensity interval training" OR "exercise	English language, January 2015–February 2025, original research articles	7-Feb-25	121	80 English-language records; 43 after title/abstract screening

	training") AND TS=("disease prevention" OR "chronic disease" OR "long-term health" OR "health outcomes")				

Searches were conducted independently by two reviewers (H.P. and R.E.) using EndNote for reference management. Duplicate records were removed using EndNote's deduplication tool, resulting in 243 unique records for title and abstract screening. Manual screening of reference lists from included articles was performed to identify additional relevant studies. After title and abstract screening, 23 full-text articles were assessed for eligibility, resulting in 11 studies included in the final meta-analysis (see Figure 1 for PRISMA flow diagram). All searches were restricted to peer-reviewed original research articles published in English between January 2015 and February 2025, excluding reviews, meta-analyses, conference abstracts, and non-peer-reviewed publications. An updated search conducted up to 01 July 2025 identified no additional eligible studies.

Table S2. Detailed Characteristics and Outcomes of Included Studies.

**Study**	**Sample Size (Intervention/Control, Gender)**	**Population (Health Status, Country)**	**Intervention**				**Primary Outcomes**	**Secondary Outcomes**	**Results**	**RoB Assessment**
			**Intervention Type**	**Duration (Weeks)**	**Frequency (Sessions/Week)**	**Intensity**				
Conroy et al., 2016	720 (320 exercisers, 400 controls; all females)	Postmenopausal women healthy, Canada	Aerobic	52	5	70–80% HRR	IL-4, IL-10	Body composition, aerobic fitness (VO2 max)	No significant differences in IL-4 or IL-10 levels between groups in either trial.<br>-<br>Circulating anti-inflammatory cytokine levels decreased during the intervention among all groups.<br>-	Some concerns

									Moderation effects were found for IL-10 but not IL-4, with differential effects by physical fitness, age, BMI, and baseline IL-10 levels.	
Niemiro et al., 2020	24 (all females)	Older women at high risk of breast cancer (USA)	Aerobic (MCT vs. HIT)	12	3	MCT: 50–60% VO2max ; HIT: 80–90% VO2max	Granulocytes, lymphocytes, CD4+ T-cells, CD8+ T-cells, CD4+ naive T-cells, CD4+ RTE, CD4:CD8 ratio, CD8+ EM cells	Cytokines (IL-6, IL-7, IL-15), β-AR expression, VO2max	HIIT decreased total granulocytes, CD4+ T-cells, CD4+ naïve T-cells, CD4+ RTEs, and CD4:CD8 ratio. - MICT increased total lymphocytes and CD8+ effector memory T-cells. - Changes in cardiorespiratory fitness (˙VO2max)	Some concerns

									were positively correlated with β2-AR expression on central memory subsets of CD4+ and CD8+ T-cells.<br>-<br>Plasma myokine levels (IL-6, IL-7, IL-15, osteonectin) did not change significantly between groups, but individual changes were related to specific lymphocyte subsets.	
Chagas et al., 2017	70 (all females)	Postmenopausal women obese [%BF ≥35%], sedentary, Brazil)	Aerobic + Neuromuscular	20	3 (75 min/session)	Aerobic: 50–60% VO2peak; Neuromuscular: Progressive stretching, isometric, dynamic exercises	IL-6, TNF-α, IL-10	IL-10/IL-6 ratio, IL-10/TNF-α ratio, BMI, WC, %BF, lean mass, BG, total	Significant reductions in TNF-α in the exercise group.<br>-<br>Increased ratio of IL-10/IL-6 and IL-10/TNF-α in the exercise group.<br>-	Low

								cholesterol, HDL-C, LDL-C, TG, VLDL-C, CK	No significant changes in IL-6 values, but a protective effect on IL-10 levels was observed. - Improvements in body composition (BMI, waist circumference, body fat percentage) and biochemical markers (total cholesterol, triglycerides, LDL-C, VLDL-C).	
Jung et al., 2022	28 (all females)	Elderly obese women with sarcopenia South Korea	Circuit train ing			60–80% HRR	Cardiovascular risk factors (heart rate, SBP, RPP, HDL-C, lipid ratios, ba-PWV, FPI, HOMA-IR),	Body composition (body weight , BMI, fat mass, fat-free mass, ASM, WHR)	Significant improvements in body composition: BMI, fat-free mass, % body fat, waist-to-hip ratio. - Cardiovascular risk factors improved:	Some concerns

							inflam-matory markers (hs-CRP, IL-6), IGF-1		heart rate, systolic blood pres-sure, rate pressure product, HDL-C, TC/HDL-C ratio, TG/HDL-C ratio, LDL-C/HDL-C ratio, bra-chial-ankle pulse wave velocity. - Inflamma-tory mark-ers de-creased: hs-CRP, IL-6.	
Ahn and Kim, 2022	39 (all fe-males)	Elderly obese women with sarcopenia, South Korea	Dy-namic Re-sistance Ex-er-cise	12	3	Adjusted via Borg's RPE scale (10–15 repeti-tions, 2–3 sets, in-creasing every 2 months)	ApoA-I, ApoA-I/HDL-C ratio, inflam-matory markers (IL-4, IL-5, IL-6, CRP, TNF-α), antioxi-dant factors (SOD2, HSP70), meta-bolic syn-drome markers	Body com-posi-tion (body weight, WHR, WC), blood pres-sure (SBP, DBP), physi-cal fit-ness (PEI, 30-s chair stand,	Significant reductions in waist-hip ratio (WHR), waist cir-cumference (WC), dias-tolic blood pressure (DBP), blood insu-lin, and HOMA-IR. - Significant increases in ApoA-I levels, ApoA-I/HDL-C ratio,	Some con-cerns

							(TG, fasting glucose, insulin, HOMA-IR)	balance test, TUG, straight walking test, S-type walking test)	SOD2, IL-4, and IL-5 levels. - Reductions in IL-6, CRP, and TNF-α levels (non-significant). - Significant increase in HSP70 levels. - Improvements in physical fitness: cardiorespiratory endurance, muscular endurance, balance, agility, and walking ability.	
Andersson et al., 2020	49 (Females: 38, Males: 11)	Older adults with rheumatoid arthritis (RA), disease duration ≥12 years, DAS28 score <3.2, Sweden	Moderate-to High-Intensity Aerobic and Resistance	20	3–4	70–89% of maximum heart rate, 70–89% of 1 repetition maximum	Regulatory immune cell populations (Tregs, Bregs, MDSCs), cytokine levels (IL-10, IL-2,	VO2 max, physical function (5 To Stand test, HAQ-DI), body mass index	Significant improvement in aerobic capacity and muscle strength. - Significant decrease in regulatory T cells (Tregs) and B cells	Low

			Exercise				IL-7, IFNγ, IL-17A, IL-22)	(BMI), blood pressure (BP), C-reactive protein (CRP), Disease Activity Score in 28 joints (DAS28), patient's global impression of change (PGIC)	(Bregs) in the exercise group, but not in the control group. - No significant changes in myeloid-derived suppressor cells (MDSCs). - Decreased serum levels of IL-10 in the exercise group. No increase in disease activity or inflammation despite reduced Treg and Breg frequencies.	
Chen et al., 2018	33 (all females)	Elderly women with sarcopenia (ASM/height² <5.7 kg/m², grip strength <18 kg), Taiwan	Kettlebell Training (Progressive Resistance Training)	8	3–4	Moderate to high, adjusted to personal capacity (8–12 repetitions, load increased if >10 reps,	Body composition (SMM, ASM, BFM, VFA, sarcopenia index), muscle strength (left and right	None explicitly defined in the study	Significant increases in appendicular skeletal muscle mass (ASM) and sarcopenia index. - Significant improvements in grip	Low

						de-creased if <8 reps)	hand grip strength, back strength), pul-monary function (PEF, FVC), chronic low-grade inflam-matory markers (hs-CRP, IL-6, TNF-α)		strength (left and right hand), back strength, and peak expiratory flow (PEF). - Retention effects ob-served for 4 weeks af-ter detrain-ing. - Significant reduction in high-sensitivity C-reactive protein (hs-CRP) lev-els. - No signifi-cant changes in IL-6 or TNF-α lev-els.	
Chupel et al., 2017	33 (all fe-males)	Older women with mild to mod-erate cogni-tive impair-ment (MMSE 10–23), institu-tionalized, Portugal	Chair-based elas-tic band strength train ing	28	2-3	OMNI per-ceived exertion scale 6–8 (some-what hard to hard), us-ing yel-low and red elas-tic bands	IL-10, TNF-α, IFN-γ, CRP, TNF-α/IL-10 ratio	MMSE (cog-ni-tion), hand-grip test, 8-Foot Up and Go Test	Significant increase in IL-10 lev-els in the ST group. - Significant decrease in leukocyte and lym-phocyte counts in	Low

								(8-FGT), 30s Chair-Stand Test (30s-CS), 30s Arm-Curl Test (30s-AC), 2m-Step Test (2m-STEP), leukocytes, lymphocytes, monocytes, granulocytes, erythrocytes, hemoglobin, hematocrit, MCV, MCH, MCHC	the ST group. - Significant improvement in Mini Mental State Examination (MMSE) scores in the ST group. - TNF-α and CRP levels significantly increased in the control group. - Positive correlation observed between changes in granulocyte counts and MMSE scores within the total sample. - Improvement in physical fitness tests including hand-grip strength, agility, and	

									lower body strength.	
Despeghel et al., 2021	40 (Males: 21, Females: 19)	Older adults, healthy and with age-typical comorbidities (e.g., hypertension, cardiovascular disease, thyroid disease), previously physically inactive, Germany	Combined low-dose resistance and endurance training (machine-supported strength circuit and cycling/cross-trainer)	6	2	Strength: 60% of 1RM, adjustable; Endurance: heart rate of 190 minus age	$CD4^+$/$CD8^+$ T cell ratio, T cell subpopulations (naïve, CM, EM, T-EMRA, $CD28^-CD57^+$)	Cytokines (IL-2, IL-6, IL-8, IL-10, VEGF, TNF-α, CCL-2, CXCL-13, ICAM-1, IL-1ra, IL-18, leptin, resistin), strength capacity (back extension, abdominal crunch, leg extension, leg flexion, chest press, seated row),	Significant increase in CD4+/CD8+ T cell ratio.<br>-<br>Significant decrease in systemic levels of IL-6, IL-8, IL-10, and VEGF.<br>-<br>No significant changes observed in total white blood cells, neutrophils, lymphocytes, or proportions of CD3+ T cells, CD4+ T cells, and CD8+ T cells.<br>-<br>Significant improvement in strength capacity after the training program.<br>-<br>Significant decrease in diastolic	Low

								anthropometrics (BMI, body fat %, visceral fat, skeletal muscle mass), diastolic BP	blood pressure in both groups. - Training group showed a significant difference in Δ-change for strength parameters compared to the control group.	
Furtado et al., 2020	60 (all females)	Frail older women with cognitive impairment (Physical Frailty Index 2.28 ± 1.32), residing in care homes, Portugal	Chair Multimodal Exercise	28	2-3	CME: 50–75% HRmax, Borg RPE 1–3 (warm-up) to 5–6 (main); CSE: low to moderate intensity with elastic bands, Borg RPE 3–6	sIgA, lysozyme, IL-6, IL-1β, IL-10, CRP, IFN-γ, TNF-α, TNF-α/IL-10 ratio	Functional fitness (Chair Seat and Reach, 30s Arm Curl, 30s Chair Stand, 2-min Step Test), anthropometrics (BMI, body mass), blood pressure	Significant decrease in TNF-α/IL-10 ratio in the multimodal exercise group. - Increase in IL-10 levels in both exercise groups compared to controls. - No significant changes in sIgA, Lys, IL-6, or IL-1β levels across groups. -	Low

								(systolic, diastolic)	Controls showed a trend toward decreased physical fitness and increased pro-inflammatory markers. - Multimodal and muscle-strength groups improved functional fitness parameters.	
Gomez-Tomas et al., 2018	38 (all females)	Postmenopausal women, no hormone therapy, no uncontrolled hypertension, diabetes, hypercholesterolemia, or cancer history, Spain	Elastic band resistance training	52	3	Progressive intensity: Phase 1 (months 1–4): OMNI-RES 3–4, yellow band (1.5 kg at 100% elongation); Phase 2 (months 5–8): OMNI-RES 5–6, red band (2 kg); Phase 3 (months 9–12):	Weight, waist circumference, total cholesterol, HDL-C, LDL-C, VLDL-C, triglycerides, CRP	None reported	Significant reductions in weight, waist circumference, total cholesterol, LDL-C, and CRP levels. - Non-significant improvements in HDL-C, VLDL-C, and triglycerides. - Control group showed significant increases in	Some concerns

						OMNI-RES 6–7, green band (2.5 kg)			weight and waist circumference. - Between-group analysis revealed significant differences in waist circumference, triglycerides, VLDL-C, and CRP levels.	

Characteristics and outcomes of the 11 randomized controlled trials included in the meta-analysis. The table details study design, sample size (intervention/control, gender), population characteristics (health status, country), intervention specifics (type, duration, frequency, intensity), primary outcomes (circulating exerkine levels: IL-6, TNF-α, IL-10, CRP, IFN-γ), secondary outcomes (e.g., body composition, cardiovascular fitness, metabolic parameters), key results, and risk of bias assessments using the Cochrane Risk of Bias 2 (RoB 2) tool. Abbreviations: IL-6, interleukin-6; TNF-α, tumor necrosis factor-alpha; IL-10, interleukin-10; CRP, C-reactive protein; IFN-γ, interferon-gamma; %BF, body fat percentage; BMI, body mass index; WC, waist circumference; VO2max, maximum oxygen uptake; HDL-C, high-density lipoprotein cholesterol; LDL-C, low-density lipoprotein cholesterol; TG, triglycerides; VLDL-C, very low-density lipoprotein cholesterol; CK, creatine kinase; hs-CRP, high-sensitivity C-reactive protein; HOMA-IR, homeostatic model assessment for insulin resistance; ApoA-I, apolipoprotein A-I; SOD2, superoxide dismutase 2; HSP70, heat shock protein 70; Tregs, regulatory T cells; Bregs, regulatory B cells; MDSCs, myeloid-derived suppressor cells; sIgA, secretory immunoglobulin A; MMSE, Mini-Mental State Examination; RPE, rating of perceived exertion; 1RM, one-repetition maximum; PEF, peak expiratory flow; FVC, forced vital capacity; ASM, appendicular skeletal muscle mass; VFA, visceral fat area; SBP, systolic blood pressure; DBP, diastolic blood pressure; TUG, Timed Up and Go test; PEI, physical efficiency index.

Table S3. Risk of Bias Assessments.

**Study**	**Randomization process**	**Deviations from intended interventions**	**Missing outcome data**	**Measurement of the outcome**	**selection of the reported result**	**Overall**
Conroy et al., 2016	Low	Some concerns	Low	Low	Low	Some concerns
Niemiro et al., 2022	Low	Some concerns	Low	Low	Low	Some concerns

Chagas et al., 2017	Low	Low	Low	Low	Low	Low
Jung et al., 2022	Low	Some concerns	Low	Low	Low	Some concerns
Ahn and Kim, 2022	Low	Some concerns	Low	Low	Low	Some concerns
Andersson et al., 2020	Low	Low	Low	Low	Low	Low
Chen et al., 2018	Low	Low	Low	Low	Low	Low
Chupel et al., 2017	Low	Low	Low	Low	Low	Low
Despeghel et al., 2021	Low	Low	Low	Low	Low	Low
Furtado et al., 2020	Low	Low	Low	Low	Low	Low
Gomez-Tomas et al., 2018	Low	Some concerns	Low	Low	Low	Some concerns

Risk of bias assessments for the 11 included randomized controlled trials using the Cochrane Risk of Bias 2 (RoB 2) tool. The table evaluates five domains: randomization process (D1), deviations from intended interventions (D2), missing outcome data (D3), measurement of the outcome (D4), and selection of the reported result (D5). Each domain is rated as low risk, some concerns, or high risk, with an overall risk of bias determined for each study. Ratings are based on independent assessments by two reviewers (H.P. and R.E.), with disagreements resolved by a third reviewer (B.K.).

Figure S1–S5: Funnel Plots

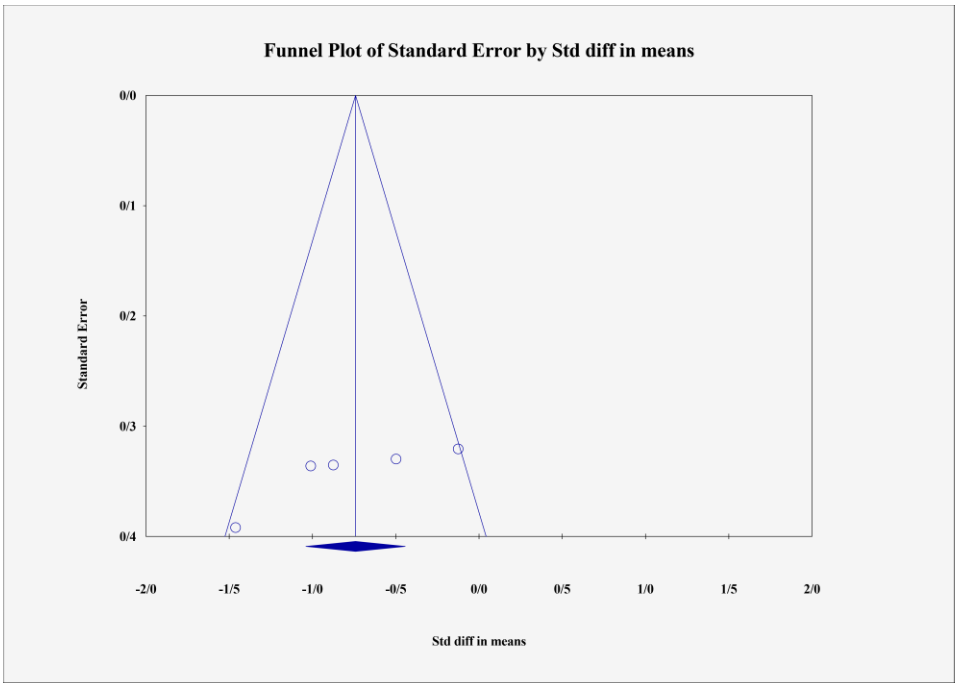

Figure 1. Funnel Plot for C-Reactive Protein (CRP). Funnel plot depicting the standardized mean difference (SMD) and standard error (SE) for studies assessing the effect of exercise interventions on CRP levels. The plot includes data from five studies [25,27,31,32]. Vertical line represents the pooled SMD (–0.77, 95% CI: –1.20 to –0.33), with dashed lines indicating the 95% confidence interval.

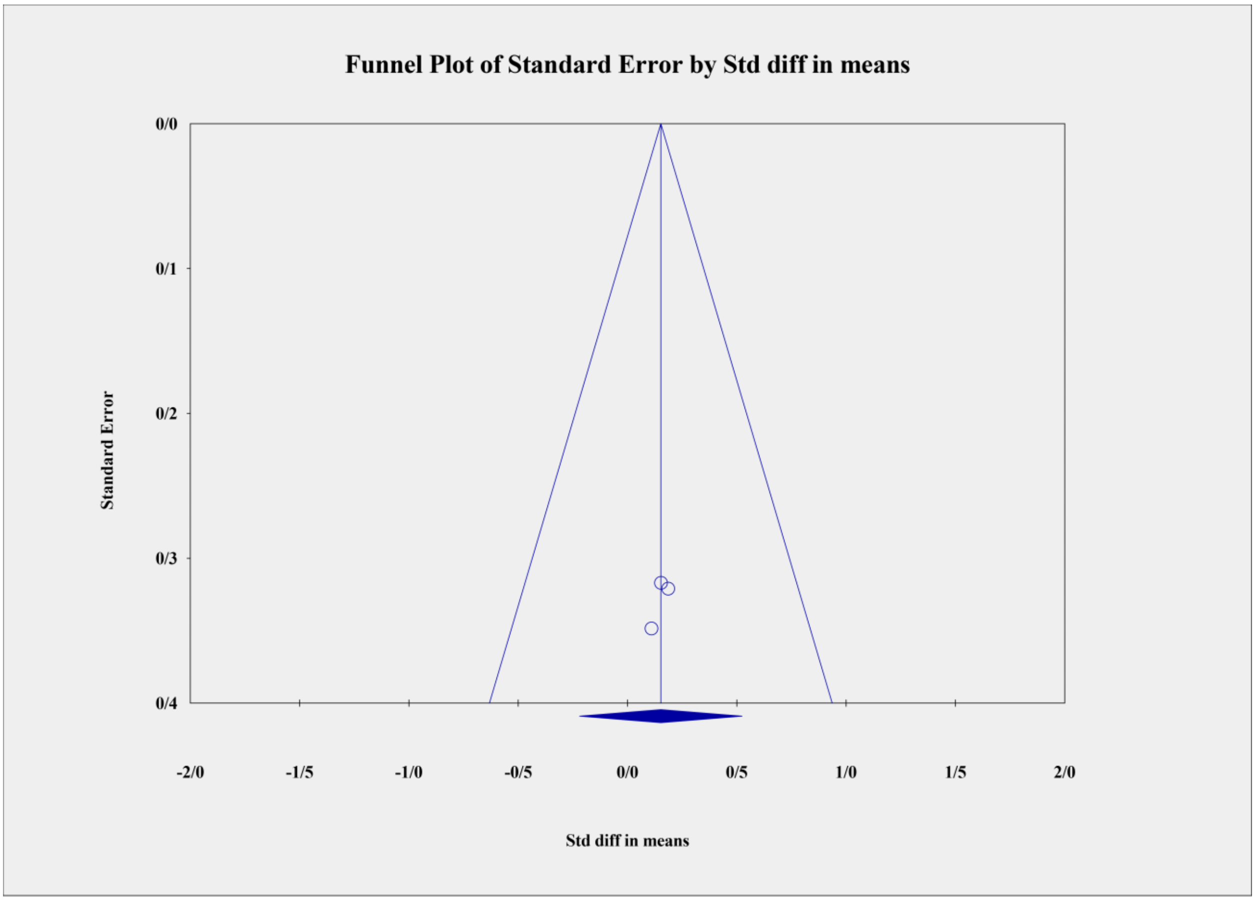

Figure S2. Funnel Plot for Interferon-Gamma (IFN-γ). Funnel plot depicting the SMD and SE for studies assessing the effect of exercise interventions on IFN-γ levels. The plot includes data from

three studies [25,27]. Vertical line represents the pooled SMD (0.15, 95% CI: –0.22 to 0.52), with dashed lines indicating the 95% confidence interval.

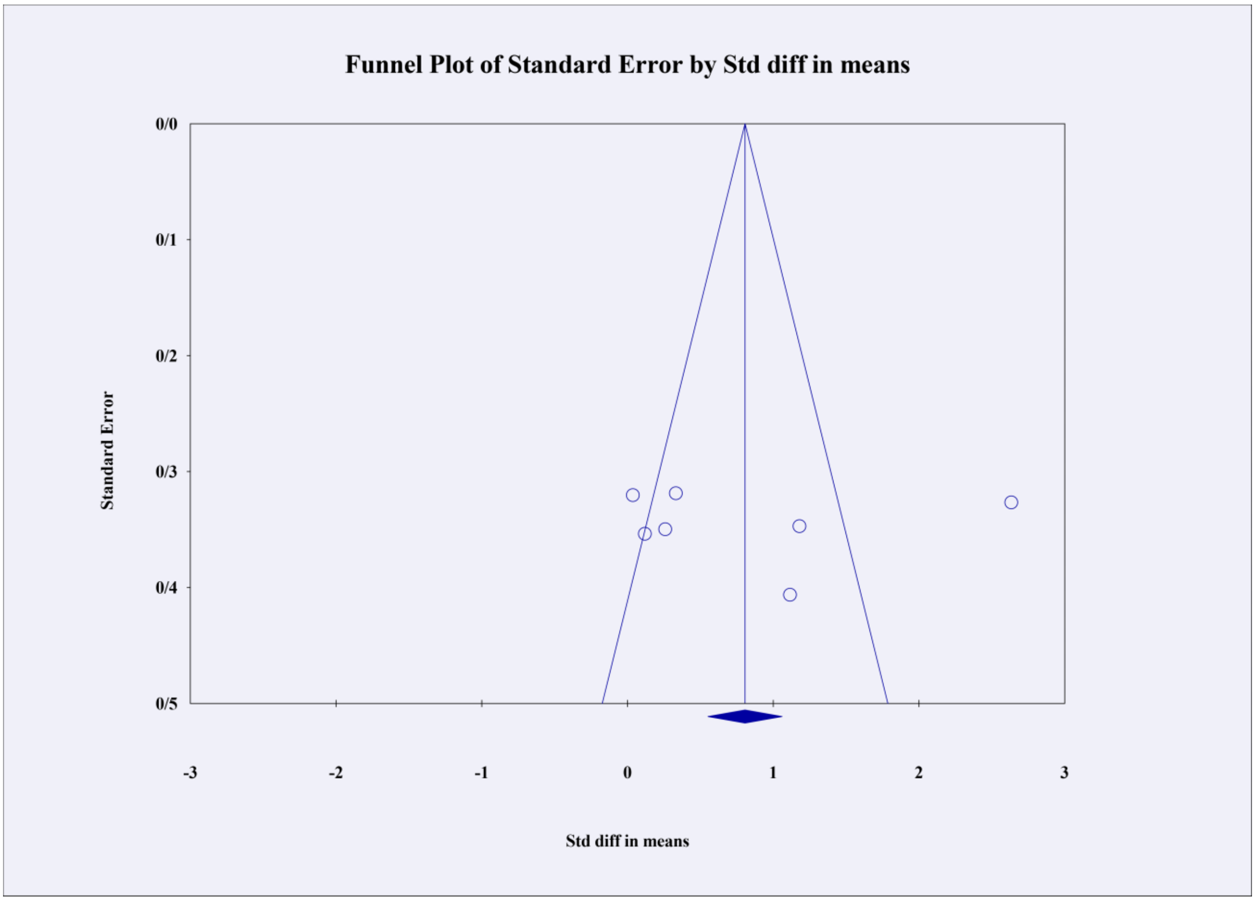

Figure S3. Funnel Plot for Interleukin-6 (IL-6). Funnel plot depicting the SMD and SE for studies assessing the effect of exercise interventions on IL-6 levels. The plot includes data from seven studies [22,26,27,30,31]. Vertical line represents the pooled SMD (0.81, 95% CI: 0.10 to 1.53), with dashed lines indicating the 95% confidence interval.

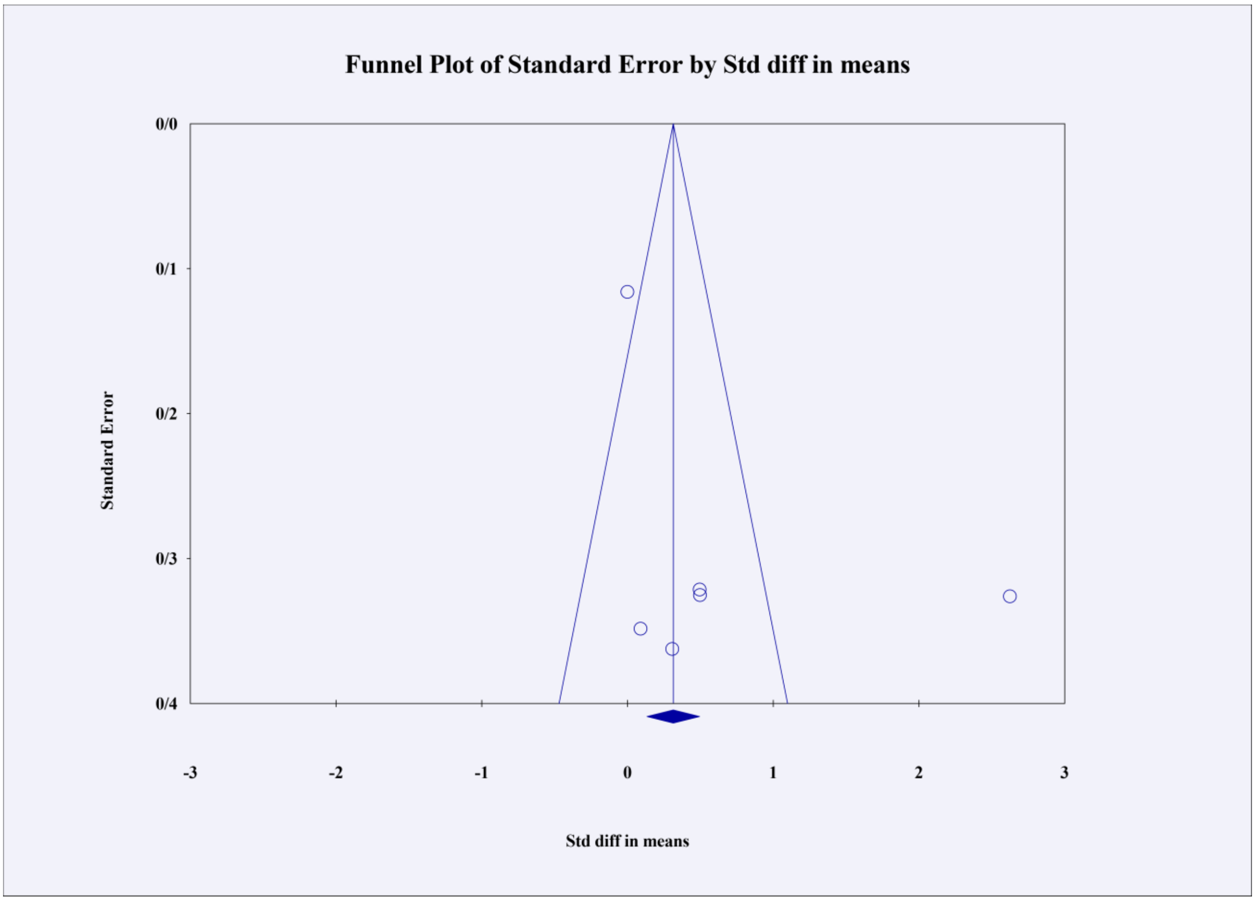

Figure S4. Funnel Plot for Interleukin-10 (IL-10). Funnel plot depicting the SMD and SE for studies assessing the effect of exercise interventions on IL-10 levels. The plot includes data from six studies [22,25–28]. Vertical line represents the pooled SMD (0.66, 95% CI: –0.09 to 1.41), with dashed lines indicating the 95% confidence interval.

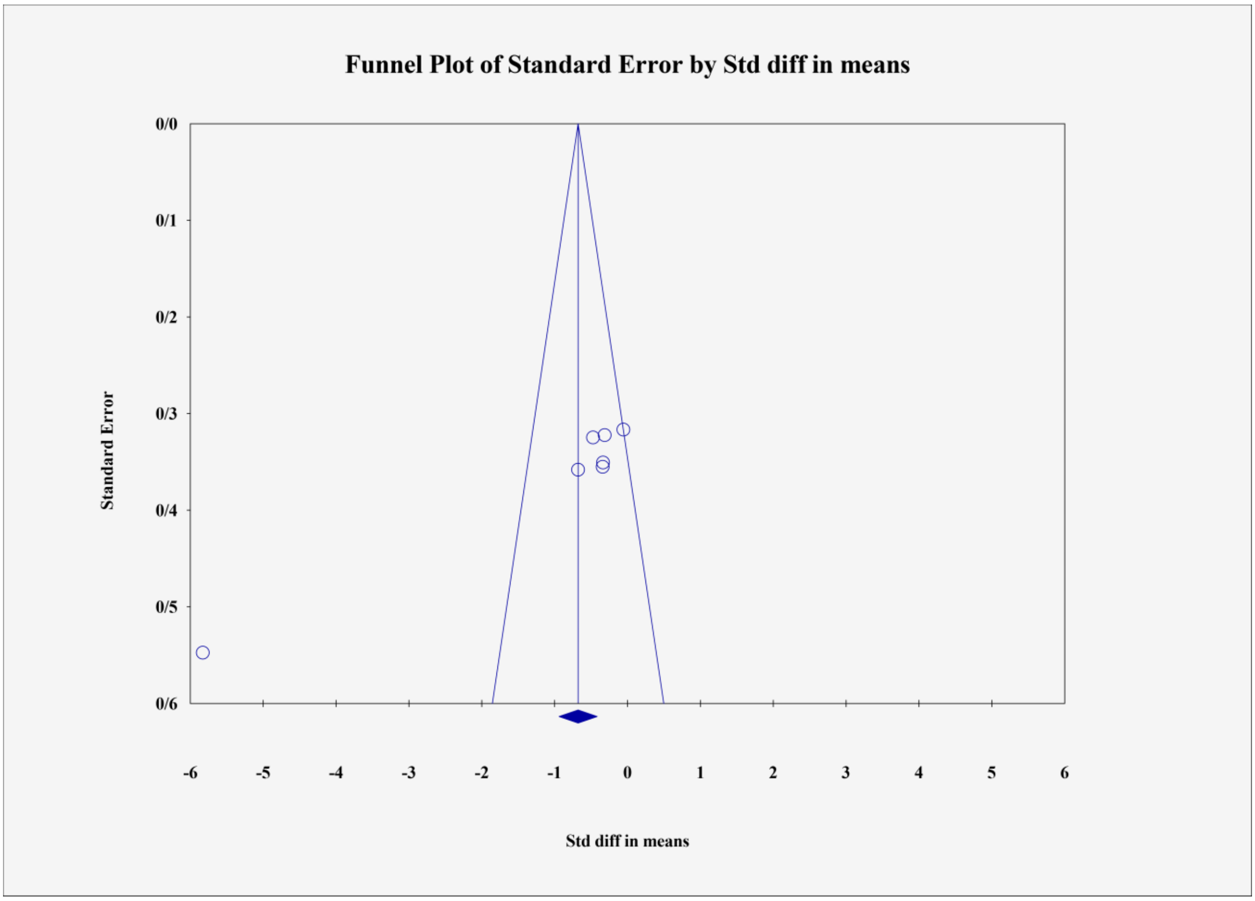

Figure S5. Funnel Plot for Tumor Necrosis Factor-Alpha (TNF-α). Funnel plot depicting the SMD and SE for studies assessing the effect of exercise interventions on TNF-α levels. The plot includes data from seven studies [22,24–27,31]. Vertical line represents the pooled SMD (–1.09, 95% CI: –2.14 to –0.03), with dashed lines indicating the 95% confidence interval.

Figures S6–S10: Sensitivity Analyses

Study name	Subgroup within study	Statistics with study removed						
		Point	Standard error	Variance	Lower limit	Upper limit	Z-Value	p-Value
Ahn and Kim, 2022	CRP	-0/927	0/191	0/036	-1/301	-0/553	-4/859	0/000
Chupel et al., 2017	CRP	-0/618	0/201	0/040	-1/012	-0/224	-3/073	0/002
Furtado et al., 2020 - CME	CRP	-0/711	0/273	0/075	-1/246	-0/175	-2/600	0/009
Furtado et al., 2020 - CSE	CRP	-0/747	0/284	0/081	-1/304	-0/190	-2/629	0/009
Gomez-Tomas et al., 2018	CRP	-0/843	0/276	0/076	-1/385	-0/301	-3/048	0/002
		-0/767	0/222	0/049	-1/201	-0/333	-3/462	0/001

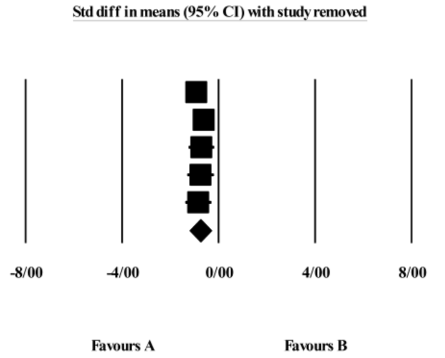

Figure S6. Sensitivity Analysis for C-Reactive Protein (CRP). Sensitivity analysis plot of SMD and SE for CRP with one study removed. Pooled SMD = –0.767 (95% CI: –1.201 to –0.333), SMDs –0.618 to –0.927 (all $p < 0.01$).

Study name	Subgroup within study	Point	Standard error	Variance	Lower limit	Upper limit	Z-Value	p-Value
Chupel et al., 2017	IFN-?	0/170	0/226	0/051	-0/272	0/612	0/754	0/451
Furtado et al., 2020 - CME	IFN-?	0/152	0/236	0/056	-0/311	0/614	0/642	0/521
Furtado et al., 2020 - CSE	IFN-?	0/134	0/235	0/055	-0/326	0/594	0/572	0/567
		0/152	0/189	0/036	-0/219	0/524	0/805	0/421

(Statistics with study removed)

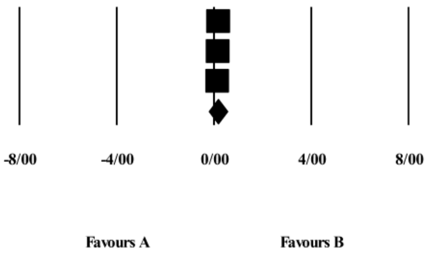

Figure S7. Sensitivity Analysis for Interferon-Gamma (IFN-γ) .Sensitivity analysis plot of SMD and SE for IFN-γ with one study removed. Pooled SMD = 0.152 (95% CI: –0.219 to 0.524), with no significant effect (p = 0.421).

Study name	Subgroup within study	Point	Standard error	Variance	Lower limit	Upper limit	Z-Value	p-Value
Chagas et al., 2017	IL-6	0/485	0/201	0/041	0/090	0/879	2/408	0/016
Jung et al., 2022	IL-6	0/762	0/418	0/175	-0/057	1/582	1/823	0/068
Ahn and Kim, 2022	IL-6	0/749	0/425	0/181	-0/084	1/582	1/762	0/078
Chen et al., 2018	IL-6	0/902	0/418	0/174	0/084	1/720	2/161	0/031
Despeghel et al., 2021	IL-6	0/925	0/410	0/168	0/122	1/728	2/258	0/024
Furtado et al., 2020 - CME	IL-6	0/892	0/425	0/181	0/059	1/725	2/098	0/036
Furtado et al., 2020 - CSE	IL-6	0/942	0/404	0/163	0/150	1/734	2/331	0/020
		0/810	0/365	0/133	0/095	1/526	2/221	0/026

(Statistics with study removed)

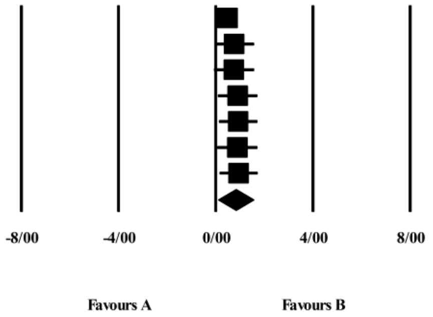

Figure S8. Sensitivity Analysis for Interleukin-6 (IL-6). Sensitivity analysis plot of SMD and SE for IL-6 with one study removed. Pooled SMD = 0.810 (95% CI: 0.095 to 1.526), with SMDs ranging from 0.485 to 0.942 (all $p < 0.05$, except specific exclusions).

Study name	Subgroup within study	Point	Standard error	Variance	Lower limit	Upper limit	Z-Value	p-Value
Conroy et al., 2016	IL-10	0/808	0/469	0/220	-0/112	1/727	1/721	0/085
Chagas et al., 2017	IL-10	0/121	0/098	0/010	-0/072	0/314	1/227	0/220
Chupel et al., 2017	IL-10	0/772	0/454	0/207	-0/118	1/663	1/700	0/089
Despeghel et al., 2021	IL-10	0/729	0/453	0/205	-0/158	1/617	1/611	0/107
Furtado et al., 2020 - CME	IL-10	0/695	0/463	0/214	-0/212	1/601	1/501	0/133
Furtado et al., 2020 - CSE	IL-10	0/694	0/461	0/213	-0/210	1/598	1/504	0/132
		0/660	0/382	0/146	-0/088	1/408	1/729	0/084

(Statistics with study removed)

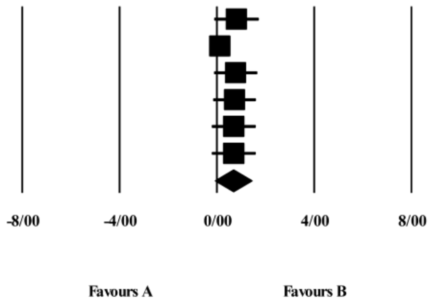

Figure S9. Sensitivity Analysis for Interleukin-10 (IL-10). Sensitivity analysis plot of SMD and SE for IL-10 with one study removed. Pooled SMD = 0.660 (95% CI: –0.088 to 1.408), with SMDs ranging from 0.121 to 0.808 ($p > 0.08$).

Study name	Subgroup within study	Statistics with study removed						
		Point	Standard error	Variance	Lower limit	Upper limit	Z-Value	p-Value
Chagas et al., 2017	TNF-a	-0/354	0/137	0/019	-0/623	-0/084	-2/572	0/010
Ahn and Kim, 2022	TNF-a	-1/204	0/646	0/417	-2/469	0/062	-1/865	0/062
Chen et al., 2018	TNF-a	-1/224	0/635	0/403	-2/468	0/019	-1/929	0/054
Chupel et al., 2017	TNF-a	-1/167	0/636	0/405	-2/414	0/080	-1/835	0/067
Despeghel et al., 2021	TNF-a	-1/223	0/633	0/401	-2/465	0/019	-1/931	0/054
Furtado et al., 2020 - CME	TNF-a	-1/273	0/634	0/402	-2/516	-0/030	-2/007	0/045
Furtado et al., 2020 - CSE	TNF-a	-1/231	0/643	0/413	-2/491	0/029	-1/914	0/056
		-1/088	0/538	0/289	-2/142	-0/033	-2/022	0/043

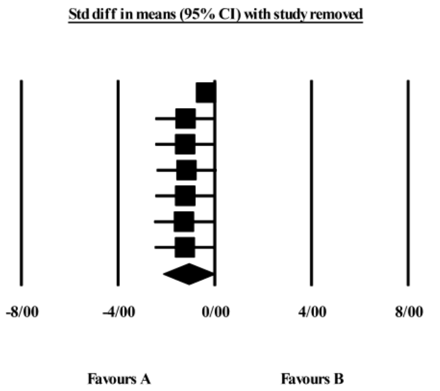

Figure S10. Sensitivity Analysis for Tumor Necrosis Factor-Alpha (TNF-α). Sensitivity analysis plot of SMD and SE for TNF-α with one study removed. Pooled SMD = –1.088 (95% CI: –2.142 to –0.033), with SMDs ranging from -0.354 to –1.222 ($p < 0.05$, except specific exclusions).
